# Neural Systems for Cognitive and Emotional Processing in Posttraumatic Stress Disorder

**DOI:** 10.3389/fpsyg.2012.00449

**Published:** 2012-10-30

**Authors:** Vanessa M. Brown, Rajendra A. Morey

**Affiliations:** ^1^Duke-University of North Carolina Brain Imaging and Analysis Center, Duke UniversityDurham, NC, USA; ^2^Mid-Atlantic Mental Illness Research Education and Clinical Center, Durham Veterans Affairs Medical CenterDurham, NC, USA; ^3^Department of Psychiatry and Behavioral Sciences, Duke UniversityDurham, NC, USA

**Keywords:** PTSD, emotion processing, cognitive control, neuroimaging, emotion-cognition interactions

## Abstract

Individuals with posttraumatic stress disorder (PTSD) show altered cognition when trauma-related material is present. PTSD may lead to enhanced processing of trauma-related material, or it may cause impaired processing of trauma-unrelated information. However, other forms of emotional information may also alter cognition in PTSD. In this review, we discuss the behavioral and neural effects of emotion processing on cognition in PTSD, with a focus on neuroimaging results. We propose a model of emotion-cognition interaction based on evidence of two network models of altered brain activation in PTSD. The first is a *trauma-disrupted network* made up of ventrolateral PFC, dorsal anterior cingulate cortex (ACC), hippocampus, insula, and dorsomedial PFC that are differentially modulated by trauma content relative to emotional trauma-unrelated information. The *trauma-disrupted network* forms a subnetwork of regions within a larger, widely recognized network organized into ventral and dorsal streams for processing emotional and cognitive information that converge in the medial PFC and cingulate cortex. Models of fear learning, while not a cognitive process in the conventional sense, provide important insights into the maintenance of the core symptom clusters of PTSD such as re-experiencing and hypervigilance. Fear processing takes place within the limbic corticostriatal loop composed of *threat-alerting* and *threat-assessing* components. Understanding the disruptions in these two networks, and their effect on individuals with PTSD, will lead to an improved knowledge of the etiopathogenesis of PTSD and potential targets for both psychotherapeutic and pharmacotherapeutic interventions.

## Introduction

Posttraumatic stress disorder (PTSD) is triggered by trauma and characterized by intrusive memories, hypervigilance, and difficulties with concentration and memory. The dysfunctions in PTSD suggest an inability of cognitive control areas in the brain to regulate affective areas, particularly in the context of trauma-related information. In this review, we examine the effects of PTSD on neural substrates of cognitive processes, with a specific focus on the interaction of cognition and emotion. We will extend an established neural model describing cognitive-emotional interactions to understand how specific regions of this network are involved in emotion processing are dissociated in response to trauma-related information. Meanwhile, understanding the neural processes underlying major symptom clusters of PTSD that also involve emotion-cognition interactions, require disease-specific models. Emotion can have opposing effects on cognition. On one hand, *emotional facilitation* of cognition occurs when emotional processing enhances cognitive speed or accuracy. On the other hand, impairment of cognitive processes may result from *emotional interference*. In PTSD, an emotional facilitation effect would permit trauma-related information to be processed faster and/or more accurately than trauma-unrelated information. Conversely, emotional interference would impair cognition when processing trauma-related information. However, emotional interference or facilitation of cognition in PTSD may extend beyond trauma-related material to trauma-unrelated emotional material. The effects of emotion on cognition will be examined to explain how attending to emotional information influences cognitive processes in individuals with PTSD. We will explore whether trauma information influences cognitive processes in the same way as emotional non-trauma information or whether trauma information is a special case of emotional information that holds privileged status vis-à-vis cognitive processes.

High intensity acute stress, as experienced during a traumatic event, sets off a cascade of neurobiological changes which initially help the body respond to acute threat. In PTSD, however, the stress response is maintained and becomes maladaptive. Changes in hypothalamic-pituitary-adrenal (HPA) axis and catecholamine function reflect the long-term effects of this stress response (Yehuda, 2006; Yehuda and Seckl, 2011). Initially, experiencing trauma increases production of norepinephrine and HPA hormones, including corticotrophin-releasing hormone (CRH), adrenocorticotropic hormone (ACTH), and cortisol. With long-term elevated activity, such as in PTSD, homeostatic feedback loops are disrupted. Although our understanding of HPA axis dysfunction in PTSD is far from complete (Yehuda, 2006), PTSD is associated with altered cortisol levels, greater reactivity to cortisol, and greater CRH concentration (Charney, 2004; de Kloet et al., 2006). Levels of norephinephrine are elevated in individuals with PTSD without cormorbid major depression (Krystal et al., 1989). Disruptions in HPA axis and catecholamine function cause subsequent alterations in other neurotransmitter systems, including serotonin (Charney et al., 1993), which precipitate large-scale modifications of brain function and structure. Several brain areas, including the hippocampus, amygdala, and anterior cingulate cortex (ACC), show reduced volume in PTSD (Bremner et al., 1995; Yamasue et al., 2003; Abe et al., 2006; Karl et al., 2006; Kitayama et al., 2006; Morey et al., 2012). Whether these changes represent a pre-existing vulnerability to PTSD or develop as a consequence of the disorder remains unclear (Gilbertson et al., 2002). These brain regions also show increased serotonin and dopamine release and turnover under stress, with accompanying decreases in cognitive ability (Murphy et al., 1996; Arnsten and Li, 2005), and may show long-term alterations in neurotransmitter function in PTSD (Krystal and Neumeister, 2009). Although current neuroimaging methodologies are limited in their ability to precisely assess neurotransmitter function, measuring the resulting alterations in brain function are useful for understanding the regional and network disruptions in PTSD.

**Figure 1:**
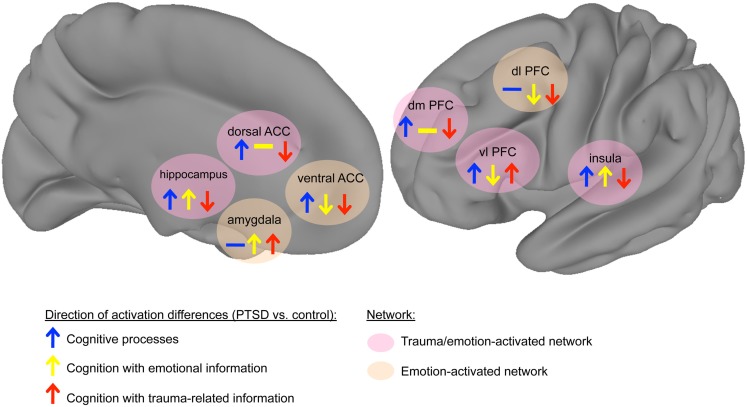
**Model of cognition-emotion interaction in PTSD**. The *trauma-disrupted* regions show differential responses to trauma-related information, reflecting attentional biases to trauma-related information in PTSD. These *trauma-disrupted network* includes the ventrolateral PFC, dorsal anterior cingulate cortex (ACC), hippocampus, insula, and dorsomedial PFC, whereas other emotion-processing regions such as the amygdala, insula, and ventral ACC appear not to be uniquely affected by trauma-related information. Arrows signify direction of activation differences in contrasting the PTSD group to the comparison group (upward arrow: PTSD > control, downward arrow: control > PTSD). Arrow colors indicate stimulus category (blue: cognition, yellow: cognition with emotional information, red: cognition with trauma-related information). Background colors of areas signify network (pink: regions differentially modulated by trauma-related information, tan: regions affected by emotion but not differentially modulated by trauma-related information). (Note: network connections are not specified because of a lack of published data describing changes in connectivity strength between nodes. Thus network connections are not explicitly shown, but implied to be consistent with known human neuroanatomy.)

Earlier neurobiological models of PTSD based on neuroimaging findings, hypothesized a hypoactive hippocampus and prefrontal cortex (PFC) that are unable to fully regulate a hyperactive amygdalar response to trauma (Brewin, 2001; Rauch et al., 2006). However, these early models reflect an understanding of neuranatomical connections with the amygdala that were elucidated primarily in rodents. Further research that elaborated amygdalar connections with the prefrontal cortical organization found in higher primates has revealed multiple, divergent roles for various regions within the PFC (Price and Amaral, 1981; Amunts et al., 2005) that process emotional information differently depending on its valence and relation to the trauma experience and memory. Our model of cognition-emotion processing put forth in this review (see Figure 1) distinguishes among multiple prefrontal areas and clarifies the roles of other brain regions based on their responses to various kinds of external information. First, we propose that specific brain regions experience unique disruptions when processing trauma-related material in PTSD. These disruptions occur within specific nodes of a more generalized emotion-processing network and an interconnected cognitive processing network. This broader emotion-processing network and its relationship to cognitive processing network has previously been described in healthy normal subjects (Yamasaki et al., 2002; Dolcos and McCarthy, 2006) as well as in major depressive disorder (Mayberg, 1997; Mayberg et al., 1999; Drevets, 2000, 2001). These models segregate attentional and emotional operations into constituent dorsal and ventral processing streams that extend into the PFC and integrate in the ACC (Yamasaki et al., 2002). In depression, the model predicts dorsal neocortical decrease in activity and ventral paralimbic increases (Mayberg et al., 1999). Our model extends the basic ventral/affective and dorsal/executive organization to specific points at which the processing of trauma-related information dissociates from emotional (trauma-unrelated) information, particularly in relation to cognitive processing.

**Figure 2:**
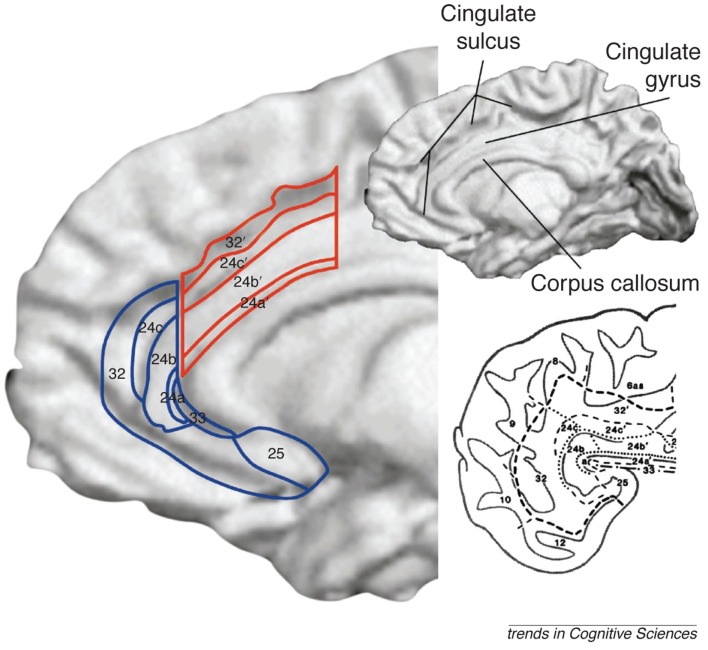
**The anterior cingulate cortex (ACC) is subdivided into the dorsal ACC (outlined in red), sometimes referred to as the mid-cingulate and the ventral ACC (outlined in blue), which can be further subdivided into the pregenual ACC (anterior to the genu of the corpus callosum) and subgenual ACC (inferior to the genu; Shackman et al., 2011)**. Ventral and dorsal streams for processing emotional and cognitive information, respectively, converge in the medial PFC and cingulate cortex. Figure from Bush et al. (2000) with permission.

During cognitive processes, the dorsal anterior cingulate and ventral prefrontal areas, including orbital frontal cortex, inferior frontal gyrus, and ventromedial PFC, show greater activation in trauma-related contexts (Morey et al., 2008b; Hayes et al., 2009; Fonzo et al., 2010). This hyperactivation is accompanied by hypoactivation in dorsal prefrontal areas, including the dorsolateral and dorsomedial PFC, as well as the hippocampus (Shin et al., 2001; Bremner and Vermetten, 2004). This model dissociates the role of specific regions to trauma-related information from their response to emotional (trauma-unrelated) material. However, other areas show alterations in their response that depend on emotional valence, not trauma content. The amygdala and insula show hyperactive responses to emotional information, but their responses to non-emotional information in PTSD have not been found to differ (Simmons et al., 2008, 2011a; Fonzo et al., 2010). The rostral/ventral ACC shows greater activation in PTSD during cognitively demanding, emotionally neutral processes and diminished activation with emotional information (Bryant et al., 2005; Kim et al., 2008; Felmingham et al., 2009a; Shin et al., 2011). For clarity, we subdivide the ACC into the dorsal ACC (red area in Figure 2) and the ventral ACC (blue area in Figure 2), which can be further subdivided into the pregenual ACC (anterior to the genu of the corpus callosum) and subgenual ACC (inferior to the genu). We propose a subnetwork within a larger emotion-processing network that exhibits altered neural responses in PTSD. These *trauma-disrupted* regions include the ventral PFC, dorsal PFC, dorsal ACC, and hippocampus, whereas other emotion-processing regions such as the amygdala, insula, and ventral ACC appear not to be uniquely affected by trauma-related information. The *trauma-disrupted regions* show differential responses to trauma-related information, reflecting attentional biases to trauma-related information in PTSD. The remaining emotion-processing regions show activation differences based on the emotional content of information rather than its relevance to trauma. This organization reflects two related dysfunctions of cognition in PTSD: difficulties processing trauma-related information and problems with processing emotional information more generally. Understanding how these two networks are altered, and how the alterations affect individuals with PTSD, will lead to an improved knowledge of the etiopathogenesis of PTSD and potential targets for therapeutic interventions.

## Cognition-Emotion Paradigms in PTSD

We have partitioned studies on cognition and emotion in PTSD into three broad categories: cognitive processing of information without emotional content, cognitive processing of information with emotional content, and cognitive processing of information with trauma-related content. The emotional or trauma information can be relevant to the cognitive process or incidental to the cognitive process, i.e., purely serving as a distractor. Specific task designs have been utilized in neuroimaging studies to probe cognition in these three broad categories. Although these tasks do not measure the same cognitive processes, they may tap into common cognitive dysfunctions in PTSD. Stimuli in purely cognitive processing consist of words, pictures, or sounds that are designed to be absent of emotion. Effects of emotional information are assessed by contrasting neutral stimuli to emotional stimuli, such as fearful faces and positive or negative pictures. In PTSD, trauma-related information is generally contrasted to neutral information. The exact content of trauma-related stimuli depends on the trauma experienced by the study subjects, but may include combat pictures or sounds for combat-traumatized subjects or angry faces for survivors of intimate partner violence. Nevertheless, a limitation of trauma-related stimuli is that stimulus-relevance cannot be exquisitely matched to individual participants’ trauma histories and results in variability of neural responses (Liberzon and Garfinkel, 2009). Contrasting the findings from these three kinds of paradigms clarifies the role of cognitive processing of neutral and emotional information in PTSD.

We review a variety of paradigms designed to assess different domains of cognition (see Table 1). This includes studies using neuroimaging methods, such as EEG or fMRI, to assess overt cognition in PTSD, including those with an emotional or trauma-related component. Behavioral studies were not included, nor were studies that involved emotional processing without explicit cognitive demands, such as fear conditioning, passive viewing or listening, symptom provocation, and script-driven imagery. While an important focus of PTSD research, these experimental designs are outside the scope of this review (for reviews see Lanius et al., 2006; McNally, 2006; Rauch et al., 2006; Francati et al., 2007; Liberzon and Sripada, 2007; Aupperle et al., 2012).

**Table 1:** **Functional neuroimaging studies of cognition-emotion interaction in PTSD**.

First author	Pub. year	Pt	TE	HC	Stimulus type	Modality	Challenge task	Amyg	vACC	Ins.	vlPFC	dACC	Hippo	dlPFC	dm-PFC
Morey	2009	22	20	–	Trauma	fMRI	Working memory retrieval	↑			↑			↓	↑
Bremner	2004	12	9	–	Trauma	PET	Stroop (emotional vs. color)			↓	↓	↓	↓	↓	
Shin	2001	8	8	–	Trauma	fMRI	Stroop (emotional vs. counting)		↓	↓	↑	↓	↑	↓	
Landre	2011	16	–	16	Trauma	fMRI	Lexical *n*-back							↑	
Morey	2008	18	21	–	Trauma/neutral	fMRI	Visual oddball		↑	↓	↑	↓		↓	
Hayes	2011	15	14	–	Trauma	fMRI	Subsequent memory	↓					↓		
Fonzo	2010	12	–	12	trauma/emotional	fMRI	Face matching	↑	↓			↑			
**Summary**								↑	↓	↓	↑	↓	↓	↓	↑
Fonzo	2010	12	–	12	Trauma/emotional	fMRI	Face matching	↑	↑	↑				↓	
Handwerger															
Simmons	2011	12	12	12	Emotional	fMRI	Face matching	↑	↓	↑					
Dickie	2008	27	–	–	Emotional	fMRI	Subsequent memory	↑					↑		
Kim	2008	12	–	12	Emotional	fMRI	Face matching with distractor		↓						
Simmons	2008	15	–	15	Emotional	fMRI	Cued anticipation			↑					
Hayes	2009	14	12	–	Trauma	fMRI	Visual oddball		↓		↓			↓	
**Summary**								↑	↓	↑	↓	Unch.	↑	↓	Unch.
Shin	2011	12	14	26	Neutral	fMRI	Motor interference					↑			
Bremner	2004	12	9	–	Trauma	PET	Stroop (emotional vs. color)		↑		↑			↑	
Shin	2007	13	13	–	Neutral	fMRI	Counting Stroop			↑		↑			
Werner	2009	12	–	12	Neutral	fMRI	Associative learning		↑	↑	↑		↑	↑	↑
Clark	2003	10	–	10	Neutral	PET	Working memory updating							↓	↑
Morey	2008	18	21	–	Trauma/neutral	fMRI	Visual oddball								
Moores	2008	13	–	12	Neutral	fMRI	Working memory updating					↓	↓	↓	
Falconer	2008	23	17	23	Neutral	fMRI	Go/NoGo			↓	↓			↓	↓
Bryant	2005	14	–	14	Neutral	fMRI	Auditory oddball	↑	↑		↑	↑		↑	
Felmingham	2009	11	–	11	Neutral	fMRI	Auditory oddball		↓			↑	↑	↑	
Sailer	2008	14	13	–	Neutral	fMRI	Decision making								↓
**Summary**								Unch.	↑	↑	↑	↑	↑	Incon.	Incon.

*TC, trauma-exposed controls; NC, non-trauma-exposed controls; Unch, unchanged; Incon, inconsistent*.

## Cognitive Processing of Non-Emotional Information

Individuals with PTSD show preserved cognition in many domains, but poorer performance during sustained attention as well as memory encoding and retrieval (Vasterling et al., 2002; Aupperle et al., 2012). During cognitive processing, our model (see Figure 1) highlights hyperactivation of ventral PFC and hippocampus and hypoactivation of dorsal PFC. The model is consistent with neuroimaging studies investigating the effects of PTSD on working memory, response inhibition, and sustained attention (see Table 1) that have established elevated activation in ventral PFC (Yamasaki et al., 2002; Bremner et al., 2004a; Bryant et al., 2005; Werner et al., 2009; although see Falconer et al., 2008 for less inferior frontal gyrus activation in PTSD). These regions are involved in emotion regulation and executive control.

Memory encoding and retrieval in PTSD is associated with greater activation in the hippocampus and parahippocampal gyrus, which also show activation in non-clinical populations during episodic memory encoding and retrieval (Shallice et al., 1994; Nyberg et al., 1996; Cabeza and Nyberg, 2000). Attention and working memory tasks that require response inhibition and monitoring show that PTSD is associated with greater activation in the pre- and postcentral gyri, which are areas typically involved in motor responses (Bryant et al., 2005; Falconer et al., 2008; Felmingham et al., 2009b; Werner et al., 2009). When processing neutral information, the downregulation of ventral PFC areas and upregulation of task-related areas in PTSD may reflect reallocation of processing resources on these task-related areas of the brain. However, results in other task-related areas are less clear. Beyond motor response areas, attention and working memory paradigms engage dorsolateral prefrontal-inferior parietal networks (Cabeza and Nyberg, 2000), which show both greater (Bremner et al., 2004a; Bryant et al., 2005; Felmingham et al., 2009b; Werner et al., 2009) and lesser (Clark et al., 2003; Falconer et al., 2008; Moores et al., 2008) activation in PTSD. Working memory has also been associated with greater activation in PTSD in the hippocampus and parahippocampal gyrus (Felmingham et al., 2009b; Werner et al., 2009), rendering the effects of PTSD on activation in these areas during cognition as inconclusive.

Cognitive processing of neutral information in the amygdala does not differ in most studies of PTSD whereas the anterior insula generally reveals increased activation (Shin et al., 2007; Werner et al., 2009; see also reduced activation in Falconer et al., 2008). Given the amygdala’s important role in emotional attention, a lack of activation for neutral information is expected. Bryant et al. (2005), found higher amygdala activation in PTSD during an auditory oddball task. This finding may have been due to a lenient region of interest analysis (criteria of *p* < 0.05, uncorrected, and three contiguous voxels), or it may reflect amygdala engagement in oddball tasks, unlike other emotionally neutral paradigms (Kiehl et al., 2005). The ACC reveals greater ventral ACC activation (Bremner and Vermetten, 2004; Bryant et al., 2005; Morey et al., 2008a; Werner et al., 2009; but see Felmingham et al., 2009b), and dorsal ACC activation (Bryant et al., 2005; Shin et al., 2007, 2011; Felmingham et al., 2009b) in PTSD. However, dorsal and ventral ACC have distinct functional roles in normative subjects, with ventral ACC active during emotion suppression and emotional conflict and dorsal ACC involved in emotional appraisal and cognitive conflict (Bush et al., 2000; Yamasaki et al., 2002; Polli et al., 2005; Etkin et al., 2011). Increased ventral ACC and decreased dorsal ACC may reflect emotional intrusion on cognitive tasks, especially when emotional distractors are intermingled with task-relevant stimuli (e.g., Morey et al., 2008b), or it may result from a disruption of normal network activity in PTSD. Dorsal ACC activation increases in PTSD resulted from Stroop and attentional tasks, whereas decreases or no differences were primarily seen in working memory or associative learning tasks, suggesting a process-specific dysfunction during attention in the dorsal ACC in PTSD; see review of Stroop findings (Hayes et al., 2012) in present issue.

A major drawback of these results is that most studies used trauma naive control participants, although a few studies (Shin et al., 2007, 2011; Falconer et al., 2008; Morey et al., 2008b) used trauma-exposed control groups. Therefore, the role of PTSD relative to trauma exposure is unclear. In general, though, these results suggest a downregulation of dorsal executive areas and an accompanying increase in activation of ventral affective areas in PTSD while performing cognitive tasks that are emotionally neutral. Thus, there is suggestion that ventral areas are tonically upregulated in PTSD even in the absence of trauma cues or other emotional information that is not trauma-relevant.

## Cognitive Processing of Emotional Information

Behavioral performance on cognitive tasks that involve emotional information (either task-relevant or task-irrelevant) has either demonstrated lack of differences in patients with PTSD (Kim et al., 2008; Fonzo et al., 2010; Simmons et al., 2011a), or lower performance with PTSD (Dickie et al., 2008; New et al., 2009). Generally, the evidence does not demonstrate a diagnosis by condition interaction where participants with PTSD perform differently only on emotional items, which would lend credence to a facilitation or interference effect. However, a behavioral study by Mueller-Pfeiffer et al. (2010) found evidence of emotional interference in PTSD. During a Stroop task, subjects with PTSD performed worse when emotional, but not neutral, pictures were shown before each trial. The findings from this study reflect intrusion of irrelevant emotional information on task performance, while most of the neuroimaging studies used stimuli with emotional content as task-relevant stimuli (Dickie et al., 2008; Simmons et al., 2008, 2011a; Fonzo et al., 2010). One neuroimaging study (Kim et al., 2008) included distracting emotional information and neutral task-relevant information, but did not find behavioral evidence of facilitation or interference.

Examining the neural differences in PTSD for emotional information shows hypoactivity in vlPFC (Hayes et al., 2009) and dlPFC (Hayes et al., 2009; Fonzo et al., 2010). In contrast, neutral material revealed dlPFC to show inconsistent and conflicting findings (see above). Our model (see Figure 1) consists of an emotion-disrupted network, comprising amygdala, dlPFC, and ventral ACC. Emotional stimuli consistently elicit greater amygdala and insula activation in PTSD (Simmons et al., 2008, 2011a; Fonzo et al., 2010). The pattern of ACC activation also differs in PTSD. The ventral ACC shows less activation (Kim et al., 2008; Hayes et al., 2009; Simmons et al., 2011a) whereas the dorsal ACC typically displays null findings (Table 1). However, increased activation was reported in a subsequent memory paradigm (Dickie et al., 2008; Fonzo et al., 2010). As with studies using neutral information, many of these studies used a non-traumatized control group, although one study used two control groups of trauma-exposed and trauma-unexposed participants (Simmons et al., 2011a).

## Cognitive Processing of Trauma-Related Information

Mirroring the findings for emotional material, neuroimaging studies using trauma-related information have rarely found that behavioral performance showed an interaction of PTSD diagnosis and stimulus type. A few behavioral studies have found such an interaction with tasks measuring attentional interference (Pineles et al., 2009) or target detection with visual, trauma-related distractors (Chemtob et al., 1999). These findings are similar to the interference effect found with emotional distractors during the Stroop task summarized above (Mueller-Pfeiffer et al., 2010). These results suggest that, if PTSD is associated with trauma-related interference, it applies to only specific cognitive processes. Specifically, the ability to disengage from task-irrelevant trauma information seems to be affected in PTSD (Chemtob et al., 1999; Pineles et al., 2009; Mueller-Pfeiffer et al., 2010; Aupperle et al., 2012), although poorer performance in PTSD may be due to the greater overall cognitive demands of interference tasks rather than an effect of interference itself. Otherwise, performance differences between trauma-related and trauma-unrelated trials have not been observed in PTSD. Contrary to the notion that PTSD facilitates performance when stimuli are interpreted as threatening, there is little evidence of better performance in PTSD with trauma-related material. In general, trauma-related information has shown either a main effect of stimulus type, with participants performing either worse (Hayes et al., 2009) or better (Hayes et al., 2011) regardless of group, or a main effect of diagnosis, with poorer performance across stimulus types in the PTSD group (Shin et al., 2001; Bremner and Vermetten, 2004; Morey et al., 2009).

Despite the paucity of behavioral findings, neuroimaging research has demonstrated that activation in a number of areas is predicted by the interaction of stimulus type and PTSD diagnosis, thus supporting the idea of a trauma-disrupted network. In our model (see Figure 1), trauma-related cognitive processing involves select trauma-disrupted regions (showing a unique response to trauma stimuli) among a generalized emotion-processing network (regions responding trauma-unrelated emotional stimuli). Generally, when processing trauma-related versus trauma-unrelated material, PTSD is associated with hyperactivity in the ventrolateral PFC, ventromedial PFC, and orbitofrontal cortex and amygdala (Morey et al., 2008b, 2009; Hayes et al., 2009; Fonzo et al., 2010), although see reports of less ventrolateral PFC activation (Bremner and Vermetten, 2004) and for less amygdala activation (Hayes et al., 2011) in PTSD. A hypoactive dorsal network, which includes the dorsolateral PFC (Shin et al., 2001; Bremner et al., 2004b), as well as lower hippocampal activation (Hayes et al., 2011). The insula, which is involved in interoceptive and affective processing, shows variable but generally greater activation with trauma-related information (Shin et al., 2001; Bremner and Vermetten, 2004; Morey et al., 2008b, 2009). These findings are concordant with studies of emotional information, which suggests the amygdala shows more activity in PTSD for emotional information regardless of the relevance to trauma. The ACC displays mixed, but generally lower ventral ACC activation (Shin et al., 2001; Fonzo et al., 2010, but see Morey et al., 2008b) and lower dorsal ACC activation (Shin et al., 2001; Bremner and Vermetten, 2004; Morey et al., 2008a) in PTSD. Compared to cognition in the absence of emotional information, studies with trauma-related information show nearly opposite patterns of activation, particularly in prefrontal regions. Specifically, when processing trauma-related information PTSD is associated with greater ventral prefrontal, lower dorsal prefrontal, greater amygdala, lower ventral ACC, and greater dorsal ACC activation. Most studies using trauma-related information have used control groups with levels of trauma similar to the PTSD subjects. The use of mostly trauma-unexposed control groups in studies with neutral information limits comparisons between these types of studies.

## Comparison of Findings Across Information Categories

Across studies using neutral, emotional, and trauma-related material, a network of brain areas shows functional differences in PTSD. The direction of effects in each area, however, depends on the type of information used. The ventral PFC, which includes medial PFC, orbital frontal cortex, and ventrolateral PFC, shows greater activation when performing cognitive processes with trauma-related material but less activation with trauma-unrelated material with emotional content. These areas are involved in a diverse array of processes, including decision making, extinction learning, and cognitive control of emotion (Bechara et al., 2000; Gray et al., 2002; Milad et al., 2007; Wager et al., 2008), but have a role in regulating affect and integrating emotional and cognitive information, and maintaining executive processes while coping with distracting affective content. Similarly, the hippocampal region shows more activation for trauma-unrelated and less for trauma-related material. These findings suggest that processing trauma-related information has a unique pattern where access to memories is modulated by connections with the amygdala (Dickie et al., 2008; Brohawn et al., 2010; Hayes et al., 2011). Conversely in the lateral PFC, trauma-related material biases the ventrolateral PFC response while reducing activation in dorsolateral areas. This pattern of activation may be related to an inability to effectively regulate trauma-induced affective responses in PTSD. In fact, individuals with PTSD show less dorsal PFC activation in PTSD when consciously up- or down-regulating responses to emotional material, suggesting that they do not recruit those areas to the same extent during emotion regulation of trauma-related material (New et al., 2009). Learning how this shift in neural processing can be corrected, either by learning cognitive strategies as in cognitive-behavioral therapy (CBT) or through guided remembering of trauma in prolonged-exposure therapy, may be a goal of research on psychotherapy for PTSD. In addition, understanding the relationships among nodes in these networks, which have been under-investigated (Gilboa et al., 2004; Fonzo et al., 2010), will explain how activation changes occur in PTSD. Examining whether these areas form connected networks or comprise parts of different networks that show similar activation patterns is an area of active research.

In contrast, the amygdala shows reduced activation in PTSD for trauma-related and trauma-unrelated emotional information. Meanwhile, studies of cognitive processing (without emotional information) have failed to demonstrate amygdala response in PTSD, suggesting that amygdala dysfunction is specific to emotional information, despite evidence from normative groups implicating the amygdala in decision making (Morrison and Salzman, 2010). It is well known that the amygdala shows activation in response to a variety of emotionally salient information, including fear, reward, and surprise (Phelps, 2006; Pessoa and Adolphs, 2010). After repeated presentations of similar stimuli, however, amygdala responsiveness decreases (Wright et al., 2001). Amygdalar habituation to emotional stimuli is attenuated in PTSD (Shin et al., 2005), leading to a sustained amygdala response even to familiar emotional stimuli. Increased amygdala activation in PTSD may reflect heightened activation to emotional content in the amygdala, lessened habituation, or both. The relationship of structural changes in the amygdala associated with PTSD to processing of emotional and trauma-related information as well as fear processing is an area that remains largely unexplored (Morey et al., 2012).

The insula is an area of interest in anxiety disorders and specifically in PTSD. The insula responds to potential threat (Simmons et al., 2008) by assigning value to incoming stimuli. In PTSD, the insula showed greater activation with emotional material and reduced activation for trauma-related material but mixed findings for neutral material. The insula is responsible for representing internal states and is involved in the anticipation of negative events (Singer et al., 2009); non-psychiatric subjects who are anxiety-prone (Simmons et al., 2006) or who exhibit faster avoidance learning (Samanez-Larkin et al., 2008) have greater insula activation. In their meta-analysis of emotion processing in anxiety disorders, Etkin and Wager (2007) found insular and amygdalar hyperactivation during fear processing. Activation in these areas represents a “final common pathway” for processing of anxiety and fear. Overactivity in the insula and amygdala to trauma-unrelated emotional material suggests that emotional information is preferentially processed through fear and anxiety networks in PTSD.

The ventral and dorsal prefrontal processing streams converge in the ACC to mediate these and other information streams from ventral limbic structures (Yamasaki et al., 2002; Dolcos et al., 2011). The dorsal and ventral portions of the ACC show similar activation patterns except for emotional trauma-unrelated stimuli. The dorsal ACC shows lower activation for trauma-related information and increased activation during cognitive processing. Meanwhile, the ventral ACC shows diminished activation for trauma related and unrelated emotional material and greater activation during cognitive processing of neutral information. Activation in the dorsal ACC, an area that may play a role in hypervigilance in PTSD (Fonzo et al., 2010), could reflect a state of high arousal and attention in PTSD that is specifically caused by trauma-related information. This finding is similar to the pattern in other trauma-disrupted areas of the brain in PTSD. Activation in the ventral ACC may exhibit greater reliance on the emotional valence of the material, rather than its relevance to trauma, although this hypothesis remains to be directly tested. The ventral ACC has connections to the amygdala and other limbic areas as well as the PFC, suggesting that this area mediates communication among emotion-related areas in the PFC and limbic system. Although the precise function of the ventral ACC is unclear, it is involved in emotional interference resolution (Whalen et al., 1998; Etkin et al., 2006) and feedback learning (Quilodran et al., 2008). In addition, increased activation in the ventral ACC correlates with symptom improvement following successful treatment of PTSD with CBT (Bryant et al., 2008) and the therapeutic response to CBT in PTSD is predicted by larger ventral ACC volume (Bryant et al., 2008). After trauma exposure, successful recruitment of the ventral ACC may mediate the attention bias for emotional information; an inability of ventral ACC to resolve emotional conflict may be involved in the onset of PTSD (Shin et al., 2001). Targeting the subgenual ACC (see Figure 2), the most ventral aspect of the ACC, with deep brain stimulation may treat refractory depression (Mayberg et al., 2005; Lozano et al., 2008), suggesting dysfunction in this region may be common to mood and anxiety disorders. Therefore, understanding and normalizing anterior cingulate dysfunction should be a vital goal in PTSD research.

Neuroimaging studies in PTSD show that patterns of brain activation differ in response to cognitive processing alone, with emotional material, and with trauma-related material. As expected by the nature of the disorder, individuals with PTSD show differences in brain activation to trauma-related information. However, their responses to other forms of emotional information also differ from people without PTSD, but only in partially overlapping ways. PTSD-related differences in brain activation during cognitive processing of trauma-related information is dissociated from processing of trauma-unrelated emotional information in *trauma-disrupted* regions including the ventrolateral PFC, insula, hippocampus, dorsal ACC, and dorsomedial PFC. Meanwhile, other regions including the amygdala, dorsolateral PFC, and ventral ACC manifest consistent differences associated with trauma-related and trauma-unrelated emotional material.

In reviewing the neuroimaging literature on PTSD that involves emotion-cognition interactions, we have enumerated empirical evidence that is largely consistent with a ventral-dorsal organization. We have highlighted regions that are dissociated based on their response to trauma-related information, into a so-called *trauma-disrupted network* as a distinct subnetwork of a larger emotion-cognition processing network. The model of interacting cognitive and emotion-processing systems elaborated by Mayberg (1997) and Drevets (2001) in depression and later in healthy normals (Yamasaki et al., 2002; Dolcos and McCarthy, 2006) is also of value in PTSD. Functional MRI studies in normative groups have established that tasks of sustained attention activate medial PFC and ACC as well as inferior parietal cortex (McCarthy et al., 1997; Kirino et al., 2000; Yamasaki et al., 2002), while those involving inhibitory behavior activate parts of the ventrolateral PFC, dorsolateral PFC, ventromedial PFC, and orbitofrontal cortex (Garavan et al., 1999; Menon et al., 2001; Aron et al., 2004). The ventrolateral PFC has been implicated in response inhibition for emotional and non-emotional settings (Compton et al., 2003; Bledowski et al., 2010). In this regard, the ACC plays a specialized role as more ventral regions are primarily involved in inhibition of responses to emotional stimuli, while dorsal regions are associated with the inhibition of neutral information (Bush et al., 1998; Whalen et al., 1998; Yamasaki et al., 2002).

The model proposed by Mayberg (1997) and Drevets (2001) provides an important foundation for understanding functional brain changes in depression (Mayberg, 1997). Mayberg’s model provides a framework for understanding the entire gamut of clinical symptoms of depression that includes cognition and attention impairments, vegetative-somatic changes (sleep, eating, and activity), and diminished mood/affect. The model segregates dorsal brain regions and ventral areas. Vegetative-somatic functions are associated with subgenual ACC (see Figure 2), anterior insula, hypothalamus, hippocampus, and brainstem. Attention-cognition functions are associated with dorsal regions including dorsalateral PFC, dorsal ACC, inferior parietal, and posterior cingulate cortex. Mood and affect changes are associated with the ventral (pregenual) ACC. As Mayberg points out, “depression is not simply dysfunction of one or another of these components, but is the failure of the coordinated interactions between the subcomponents of either compartment and between the two compartments.” Overall, the model predicts dorsal neocortical decrease in activity and ventral paralimbic increases in depression (Mayberg et al., 1999).

While there are specific aspects of this model that are clearly relevant to PTSD, particularly understanding cognitive-emotional interactions, the model does not adequately comprehend the effect of traumatic events and trauma-related information as distinct from emotional (trauma-unrelated) information. Moreover, this comparatively narrow treatment of cognitive-emotional processing does not encompass major symptom clusters of PTSD such as re-experiencing (frequent memories and thoughts of the trauma, reliving the trauma), hyperarousal (being frequently on guard, hyperalert, suddenly startled), and avoidance of persons and places that trigger reminders of the trauma (reviewed in Davidson et al., 1997; McDonald et al., 2008, 2009; Hayes et al., 2012). A traumatic experience is classified as Citerion-A of DSM-IV but is not a required diagnostic feature of depression, which makes it unique to PTSD (First et al., 1997). While depression may be precipitated by life stressor(s) (e.g., job loss, divorce, etc.), these stressful events are fundamentally different from a traumatic events. Moreover, onset of a depressive episode is common even in the absence of any environmental precipitant (Kessler, 1997).

The model put forth by Drevets (2001) postulates abnormal activity in the amygdala, ventral ACC, the orbitofrontal cortex, ventrolateral PFC, dorsomedial PFC, dlPFC, anterior insula, ventral striatum, posterior cingulate cortex, and thalamus where activity in regions that mediate emotion and stress respond with elevated activity and regions mediating attention and sensory processing respond with reduced activation. Our model simply extends the basic ventral/affective and dorsal/executive organization to include the processing of trauma-related information processing, which occupies a privileged position that is neurally dissociated from emotional (trauma-unrelated) information, particularly in relation to cognitive processing. To put the present information on cognitive-affective processes into the larger context of a model that reflects the neural alterations associated with the full syndrome of PTSD, we review a network model that explains key PTSD symptom features, particularly re-experiencing and hypervigilance.

## Cognition-Emotion Interactions in Fear Learning and Extinction

Associative fear learning, while not a cognitive process in the traditional sense, provides important insights into the maintenance of the core symptom clusters of PTSD such as re-experiencing and hypervigilance (Jovanovic et al., 2009; Jovanovic and Ressler, 2010; Norrholm et al., 2011; Mahan and Ressler, 2012). Fear learning is typically adaptive in that danger is signaled by the similarity of current threat cues with previously encountered conditions predictive of aversive outcomes (Blechert et al., 2007). However, the transfer of fear to innocuous stimuli following a traumatic experience can lead to maladaptive consequences and marked impairment of functioning in occupational and social domains (Amaya-Jackson et al., 1999). For instance, trauma-exposed individuals who widely cast defensive behaviors toward a broad range of stimuli are at risk of wasting energy resources that compromise cognitive functions and promote anxiety (Dunsmoor et al., 2011).

Extensive psychophysiological and neurobiological research in both humans and non-human animals has established several key regions involved in fear learning processes, including the amygdala, insula, cingulate gyrus, striatum, sensory cortex, and PFC (Phelps and LeDoux, 2005). Pavlovian fear conditioning and extinction of an acquired fear response has been the focus of several behavioral experiments in PTSD (Debiec and LeDoux, 2006; Blechert et al., 2007; Milad et al., 2008; Shin and Handwerger, 2009; Norrholm et al., 2011). However, extremely few studies to date have examined fear processing in the neuroimaging setting (Bremner et al., 2005; Milad et al., 2009; Rougemont-Bücking et al., 2011; van Well et al., 2012; reviewed in Hayes et al., 2012). Therefore, much of our review on the network organization of fear learning systems is extrapolated from neural models derived from healthy subjects as well as non-human primates and rodents. Pavlovian fear conditioning is a relevant model for PTSD where learned fear may persist for years and sometimes a lifetime after trauma exposure(s). Purportedly, the exaggerated and persistent fear responses to reminders of the initial psychological trauma in PTSD are associated with impairment in the extinction recall memory (Milad et al., 2009).

A basic neural circuit model that is at the basis of re-experiencing and hyperarousal symptoms of PTSD can be partitioned into two main circuits that include the temporo-striatal and corticostriatal circuits, sometimes considered together as the limbic corticostriatal loop (Cardinal et al., 2002). The general organization of the loops has been worked out in the context of Pavlovian conditioning in animal models and to some extent in humans as well (Cardinal et al., 2002). The system can be visualized as concentric loops passing through the striatum and the multiple cortical association regions. The major components of this loop are the (i) medial temporal lobe structures, including the hippocampus, amygdala, and extended amygdala regions such as the bed nucleus of the stria terminalis; (ii) striatal structures, including the caudate nucleus; (iii) medial frontal regions, including the ventromedial PFC, ACC, dorsomedial PFC, and precuneus; and (iv) lateral prefrontal structures, including the insula and ventrolateral PFC.

To understand this model in the context of PTSD, a logical starting point is hippocampal function which is necessary to access episodic memory for comparing current information to past experiences (Iordanova et al., 2009). The hippocampus can construct an encoded memory that is the conjunction of spatial and contextual information and other details about threat-related cues when functioning effectively. On the other hand, partial encoding may result in fractured memory that is inaccurate and lacking detail (see Mickley Steinmetz et al., 2012 in present issue). In PTSD, there is inadequate input from the hippocampus or a predilection toward gist based information (Brewin et al., 2010; Hayes et al., 2011). Consistent with this, PTSD patients have pronounced volume loss of the hippocampus (Karl et al., 2006; Morey et al., 2012) particularly in the dentate gyrus (CA3; Wang et al., 2010), which is essential for intact contextual memory (Nakashiba et al., 2008). Hippocampal dysfunction can bias learning strategies toward discrete associations between environmental features and the traumatic event, which in PTSD may bias fear learning and recall toward simple discrete item associations over contextual associations (Iordanova et al., 2009; Rudy, 2009). The medial temporal lobe provides inputs to the striatum and the ACC for computing an error signal between a predicted and observed outcome to indicate the need for deployment of attentional resources in order to adjust behavior or cognition (Botvinick et al., 2004). This information is resolved against potentially conflicting information and is selectively attended by the ACC (Shin et al., 2001; Hayes et al., 2009) and striatum. The striatum is strongly implicated in learning reinforced by both rewarding and aversive outcomes (LaBar et al., 1998; Phelps et al., 2004). Likewise, this information is placed in the appropriate semantic and autobiographical context through connections to dorsomedial PFC and the precuneus, which contribute the extent of self-relevance and self-reference. Striatal and medial PFC structures inform the ventrolateral PFC to maintain cognitive control over emotional distraction while assessing potential threat relevance.

In parallel, the amygdala and vmPFC are responding to potential threat and ensuing fear. The vmPFC is critical in learning about fear and safety cues, and lesions in this region produce impairment in extinction retention (Milad and Quirk, 2002; Phelps et al., 2004). The insula also responds to potential threat (Simmons et al., 2008) by assigning value to the input stimulus. The insula has been studied intensively in relation to anticipatory reward processing but there is growing evidence that it plays an analogous role in anticipatory signals important for learning about aversive outcomes (Paulus et al., 2003; Paulus and Stein, 2006; Delgado et al., 2008; Somerville et al., 2010). The inputs from the insula to the striatum may be important for responding to stimuli that share properties with a learned threat in order to adaptively react to potential threats from the environment (Delgado et al., 2008). Finally, inputs from the medial PFC, striatum, and insula are being integrated in the ventrolateral PFC to facilitate cognitive control and subsequently in the dorsolateral PFC to place information within the context of current priorities and plans.

In summary, this neural model can be conceptualized as having a *threat-alerting* component that consists of the amygdala, insula, and vmPFC, and a *threat-assessing* component that consists of hippocampus, anterior cingulate, striatum, dorsomedial PFC, precuneus, and ventrolateral PFC. A functional balance between the threat-alerting and the threat-assessing systems following trauma exposure facilitates a highly resilient response, whereas as an imbalance can result in PTSD symptoms. In PTSD the neural system is biased in favor of activation in the *threat-alerting* system over the *threat-assessing* system.

## Future Directions

Examining both trauma-related and emotional trauma-unrelated emotional material within a single study design are uncommon (Morey et al., 2008b; Fonzo et al., 2010). Furthermore, these categories have not been compared directly even when contained within the same study. Understanding how individuals with PTSD react to emotional material with or without trauma reminders is necessary to develop an accurate model of cognition and emotion in PTSD that will inform the design of more effective treatments. Future studies should directly contrast these stimulus categories. Most neuroimaging studies included emotional or trauma-related stimuli in the cognitive process of interest, instead of using such stimuli as distractors. A comparison of task-relevant and distracting information may clarify the mechanisms of emotion or trauma-related interference or facilitation in PTSD.

The emotional and trauma-related information from some studies was relevant to the ongoing task whereas in other studies it was irrelevant to the ongoing task (served only as a distractor). For instance studies of Stroop tasks (Shin et al., 2001; Bremner et al., 2004b) or episodic memory (Dickie et al., 2008; Brohawn et al., 2010; Hayes et al., 2011) that examined neural response to encoding necessarily employed task-relevant stimuli (reviewed in Hayes et al., 2012). On the other hand other types of tasks such as working memory (Morey et al., 2009) or the oddball task (Hayes et al., 2009) utilized task-irrelevant information to distract participant from the cognitive demand of the ongoing task. It is unclear how these types of differences might modulate the response in cognitive, emotion, or trauma processing networks until specific comparison studies are performed.

Few studies have examined connections among different areas in the networks. Among the studies we reviewed, two studies that explored network connectivity found amygdala connectivity differences in PTSD with the insula, ventral ACC, and ventrolateral PFC (Fonzo et al., 2010; Simmons et al., 2011a). A few additional studies that lack cognitive processing have examined connectivity relationships in response to symptom provocation (Gilboa et al., 2004) and rest (Rabinak et al., 2011; Sripada et al., 2012). More studies assessing connectivity across cognitive, emotion, and trauma processing networks are required to determine how these brain networks are related.

By incorporating recent findings, our model provides a finer-grained survey of brain areas involved in PTSD that move beyond previous models consisting of ventromedial PFC amygdala, and hippocampus. However, activation patterns in this model for regions such as ventrolateral PFC and insula, are composed of several regions that differ anatomically and functionally. Areas such as the amygdala and hippocampus also contain a number of functionally heterogeneous subregions (Amunts et al., 2005). Studies that successfully dissociate these areas will offer a more nuanced view of neural dysfunctions in PTSD.

A handful of studies on cognition-emotion interactions have begun to apply findings to improve understanding of the etiology and treatment of PTSD. Behavioral and neural performance on a motor interference task in twins discordant for combat exposure revealed that trauma-unexposed twins performed similarly to their co-twins with PTSD, despite the lack of a PTSD diagnosis (Shin et al., 2011). This finding, combined with higher pre-trauma IQ as a resilience factor (Buckley et al., 2000; McNally, 2006), suggests that the deficits reviewed above may be due to pre-existing vulnerabilities. Further research examining cognition-emotion interactions before and after developing PTSD or in twin pairs will clarify deficits reported as pre-existing vulnerabilities versus deficits that develop because of PTSD. Neuroimaging of cognitive-emotional processing tasks may hold value in improving and tailoring treatments for PTSD (Bryant et al., 2008).

Existing studies on the interaction of cognition and emotion in PTSD are beginning to coalesce on the roles of specific brain areas, but findings are still inconsistent and unclear. Improving a few key methodological considerations will clarify the neural disruptions in PTSD. First, attributing the differences to PTSD rather than trauma exposure will be simplified by using control groups matched for level of trauma exposure. Alternatively, including two control groups, trauma-exposed and -unexposed, will dissociate the differential effects of trauma and PTSD. This study design will also enable investigation of resilience factors in trauma survivors who do not develop PTSD. Second, many of the studies we reviewed had small sample sizes, a frequent limitation of neuroimaging studies. Better planned, coordinated, and analyzed studies with larger sample sizes will improve statistical validity and provide more definitive results that are less prone to false positive results (Palmer, 2000; Simmons et al., 2011b; Yong, 2012).

Conventional task-based fMRI analyses in patient populations are usually conducted by examining the interaction of stimulus type and diagnosis (e.g., trauma-related versus neutral information in PTSD versus control subjects). Although this approach addresses many methodological issues in fMRI analysis, concordant behavioral findings were not reached in many of the studies we reviewed. Indeed, most studies lacked evidence that behavioral performance showed interaction of stimulus type by diagnosis, yet they reported an interaction for the corresponding neuroimaging findings. Another issue with this analysis setup is the necessity of relative, rather than absolute, baselines in fMRI. The conventional approach of contrasting emotional with neutral stimuli, first within and then between subjects, may not capture the nature of the neural differences. As an example, an emotionally neutral attention task may find less activation in ventral ACC in PTSD when contrasting attentional tasks with a non-attentional baseline. When examining emotion processing, the same neutral attentional task may be contrasted with an emotional attentional task, and a greater difference in ventral ACC activation is found in PTSD. However, it is unclear if this larger activation difference is due to lower activation during the neutral attention task or to greater activation during the emotional task. A focus on correlating behavioral effects with differences in neural activations, more sensitive task paradigms such as parametric modulation studies, and the use of neuroimaging techniques that go beyond cognitive subtraction to study network-based connectivity, will improve the relevance of neuroimaging findings to behavioral dysfunction in PTSD.

Despite finding significant neural differences in PTSD while processing information with emotional or trauma-related content, few studies found corresponding behavioral differences. The behavioral components of these generally found a main effect of stimulus type or a main effect of PTSD diagnosis, but failed to find an interaction effect. However, a significant interaction of stimulus type by PTSD diagnosis has generally been reported by purely behavioral studies (lacking a neuroimaging component). Several reasons for this discrepancy are possible. First, the addition of neuroimaging may impose restrictions on the complexity and sensitivity of task design and/or analysis methods; the lack of behavioral findings may reflect the difficulties of teasing apart small behavioral effects in paradigms ill suited for the fMRI, PET, or EEG environment. Second, the lack of behavioral differences in the setting of corresponding neural findings indicates that the task is insufficiently sensitive to detect differences between groups that become evident only when probing the underlying neural processes. Third, the neural effects may reflect successful compensatory efforts by participants with PTSD who are able overcome behavioral deficits.

In conclusion, behavioral evidence of emotional facilitation or interference of cognition is sparse in PTSD. However, individuals with PTSD have difficulty withdrawing attention or shifting attention away from emotional information, particularly when the information is trauma-related. During emotion-related cognitive processes, individuals with PTSD show altered neural responses in a number of brain regions, which can be grouped into a trauma-disrupted and an emotion-disrupted network. These networks show that although trauma-related material has unique effects on brain activation in PTSD, the effects of emotion processing on cognition are not limited to trauma-related information. Elucidating how these areas differ through direct comparison and how the neural differences in PTSD can be addressed by psychotherapy and pharmacotherapy will improve our understanding and treatment of PTSD.

## Conflict of Interest Statement

The authors declare that the research was conducted in the absence of any commercial or financial relationships that could be construed as a potential conflict of interest.
